# Improving the Performance of Steel Machining Processes through Cutting by Vibration Control

**DOI:** 10.3390/ma14195712

**Published:** 2021-09-30

**Authors:** Mihaela Oleksik, Dan Dobrotă, Mădălin Tomescu, Valentin Petrescu

**Affiliations:** Faculty of Engineering, Lucian Blaga University of Sibiu, 550024 Sibiu, Romania; mihaela.oleksik@ulbsibiu.ro (M.O.); tomescumadalin88@yahoo.com (M.T.); valentin.petrescu@ulbsibiu.ro (V.P.)

**Keywords:** steel machining processes, cutting, tools, vibration control, roughness analysis

## Abstract

Machining processes through cutting are accompanied by dynamic phenomena that influence the quality of the processed surfaces. Thus, this research aimed to design, make, and use a tool with optimal functional geometry, which allowed a reduction of the dynamic phenomena that occur in the cutting process. In order to carry out the research, the process of cutting by front turning with transversal advance was taken into account. Additionally, semi-finished products with a diameter of Ø = 150 mm made of C45 steel were chosen for processing (1.0503). The manufacturing processes were performed with the help of two tools: a cutting tool, the classic construction version, and another that was the improved construction version. In the first stage of the research, an analysis was made of the vibrations that appear in the cutting process when using the two types of tools. Vibration analysis considered the following: use of the Fast Fourier Transform (FFT) method, application of the Short-Time Fourier-Transformation (STFT) method, and observation of the acceleration of vibrations recorded during processing. After the vibration analysis, the roughness of the surfaces was measured and the parameter Ra was taken into account, but a series of diagrams were also drawn regarding the curved profiles, filtered profiles, and Abbott–Firestone curve. The research showed that use of the tool that is the improved constructive variant allows accentuated reduction of vibrations correlated with an improvement of the quality of the processed surfaces.

## 1. Introduction

Most parts in the machine building industry are also made by using certain machining processes by cutting. Under these conditions, it is necessary to optimize the machining processes by cutting, so as to achieve a reduction in manufacturing costs and determine an increase in the life of the products made. In this context, it is necessary to develop new cutting tools with superior characteristics or better performance than the existing ones. The cost of cutting tools currently used in the manufacturing industry is high and significantly influences the total manufacturing cost, and, consequently, the final price of the product. The cost of tools can be associated not only with the cost of manufacturing tools, but also with their durability, which influences the productivity of processing, but also the quality and accuracy of processed surfaces [1,2]. The surface quality of a workpiece is given, in generally accepted terms, by the surface roughness, the most common being the average roughness Ra [3,4].

In the turning process, the most important parameters that influence the roughness of a surface are the feedrate and tool nose radius [5]. However, due to the complex structure of turning processes, there are also a number of dynamic parameters that can influence the roughness of surfaces [6]. During machining, vibrations occur due to the lack of rigidity of the machine tools and the way the cutting tools are fixed or due to changing processing conditions during the machining process. Vibrations are, generally, undesirable phenomena that can adversely affect the cutting process. Thus, during the relative movement of the cutting tool and the workpiece, undesirable results occur due to the presence of vibrations, such as increased wear of the cutting tool, and also non-compliance with the geometric accuracy of the quality of the workpiece surfaces [7].

However, there are situations in which machining by turning of parts, using controlled vibrations, caused by ultrasonic activation of the cutting tool, generates an improvement in cutting conditions, resulting in the removal of material addition, both by mechanical effect and cavitation [8,9]. Consequently, in the presence of ultrasonic vibrations, the mechanism of material removal is fundamentally changed due to the change of the cutting characteristics [10,11].

The new technologies for the manufacture of tool materials, but also those to be processed (titanium alloys, composite materials, etc.) require the improvement of traditional cutting processes using a hybrid manufacture that consists of combining precision machining with tools vibration (or workpieces) with high frequency and low amplitude [12,13]. Vibration control during machining processes can generate lower average machining forces and thinner chips, leading to high machining efficiency [14], better tool durability [15], better quality of the surface and a precise shape, and a reduction of the generation of burrs in the cutting process [16,17].

Vibration-assisted processing has huge potential in producing textured surfaces and improving the processing performance. However, the widespread application of ultrasonic activation of machining processes by cutting still has challenges determined by correctly understanding the influence of vibration on surface quality [18]. Additionally, some issues are not fully known, such as determining the conditions under which ultrasonic vibration systems could improve the efficiency of electromechanical conversion and determining methods to ensure that the working frequency during processing is stable and close to the frequency of resonance [19,20,21]. For the analysis of the vibration phenomenon that occurs during the machining processes, a method of monitoring the magnitude of the forces during the cutting process must be considered. In this sense, the interval method can be used to quantify the uncertainty, which saves time, compared to the probability method and of the statistics. Additionally, the application of the interval method allows a quick identification of the causes that determine the occurrence of vibrations and their diagnosis [22,23]. The presence of vibrations during the cutting processes influences the amount of heat released and, implicitly, the temperature of the tool of the part material. Thus, for monitoring the heat released, modern methods can be applied that involve the use of heat dissipation algorithms that allow good results in terms of the temperature distribution to be obtained [24,25].

Additionally, the use of ultrasound in cutting processes is less competitive on the real market due to the equipment, which must be used in an industrial setting (ultrasonic generator, transducer, etc.). In these conditions, it is necessary to find new constructive solutions for the cutting tools that allow control of the vibrations that appear in the cutting processes. Currently, the tools used in manufacturing processes by cutting are made of rigid elements that cannot allow control over vibrations. It should also be noted that the designed geometry of the tools used in machining is a constructive geometry and not a functional one. Very often, during machining by cutting, the functional geometry of the tool differs from the constructive geometry, and this causes an uncontrolled vibration.

Based on the above, the research presented in this paper aimed to identify the possibilities of making a cutting tool that has an optimal functional geometry depending on the processing conditions and allow the control of the vibrations that appear in the process of processing by cutting. In this sense, the research was mainly focused on the operations of turning with a transverse advance of large diameter parts. This variant of machining was chosen because the functional geometry of the tool is greatly influenced by the diameter to be machined. Thus, the research aimed to establish how the use of a tool with variable geometry allows a reduction of vibrations in the cutting process to be obtained in the sense of their control, with positive effects on the roughness of the processed surface. The paper is structured in the following sections: Materials and methods, results and discussions, and conclusions.

## 2. Materials and Methods

### 2.1. Materials Used in Research

For carrying out the experimental research, cylindrical samples with a diameter Ø = 150 mm made of C45 steel (1.0503): EN 10277-2-2008 were used. The decision to use this steel in research was made due to the fact that it is widely used in the manufacture of parts in the machine building industry. Regarding the composition of C45 steel, it is presented in Table 1, and the mechanical properties in the normalized state are shown in Table 2.

### 2.2. Equipment and Tools Used in Processing by Cutting

**Figure 1:**
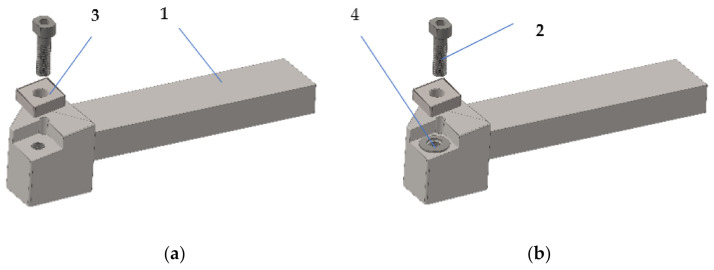
Tools for front turning: (**a**) in the classic version (T01); (**b**) with an improved constructive shape (T02), 1—the body of the tool; 2—screw of fixing; 3—removable plate, 4—elastic washer.

Technological equipment, such as a numerically controlled lathe type, produced by the lathes plant in Arad, Romania, was used for the experimental research. The parameters of the machining process were *n* = 500 rpm, machining depth a_p_ = 1 mm, and transverse feed f = 0.36 mm/rev. The machining process was performed using a knife for front turning, as shown in Figure 1. This knife is characterized by the fact that it consists of a body with a special construction, with the section of the knife body q = 20 × 30 mm^2^ and a removable plate CCGT code provided by Seco Tools, Brașov, Romania.

The knife assembly thus obtained is characterized by the fact that it has a constructive geometry characterized by a clearance angle γ = 8°, seating angle α = 6°, and main angle of attack χ = 92°.

**Figure 2:**
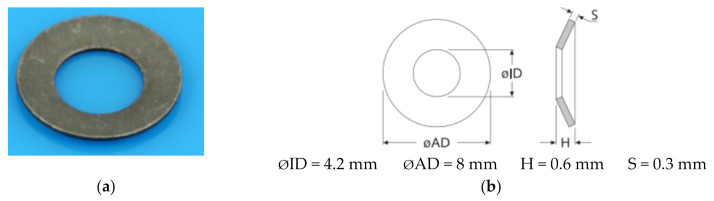
Spring washer, disc spring, DIN 2093 B. (**a**) image of spring washer; (**b**) the dimensions of the spring washer.

The process of front turning is quite complex, especially in the case of large diameter parts, and this is determined by the fact that the functional geometry of the tools changes during machining. Thus, the functional geometry changes in the sense that the clearance angle γ increases, and the seating angle α decreases with the reduction of the value of the machined diameter. For this reason, during the research, a constructive variant of the knife was proposed, as shown in Figure 1b, which would allow a functional geometry as close as possible to the optimal value to be obtained. The improved variant, as shown in Figure 1b, involved the insertion of an elastic washer under the removable plate, as shown in Figure 2, so that the functional geometry of the tool is maintained at values as close as possible to the optimal value. The presence of the spring washer allows a permanent positioning of the tool plate, depending on the processing conditions, to ensure an optimal functional geometry. By ensuring an optimal functional geometry, a reduction of the frictions that appear during the cutting is obtained and, thus, the forces but also the vibrations will have lower values. The choice of the spring washer must be made according to the processing conditions by cutting but also according to the processed material. Under these conditions, the cutting tool with improved construction can be used for processing various types of material and various cutting processes, provided that the appropriate spring washers are chosen. The spring washer and disc spring, as shown in Figure 2, comply with DIN 2093 B, A2 1.4305 steel, and are manufactured by Vinsco Spring Limited, Changzhou, China.

### 2.3. Analysis of the Evolution of the Functional Geometry of the Tool at Turning with Transverse Feed 

**Figure 3:**
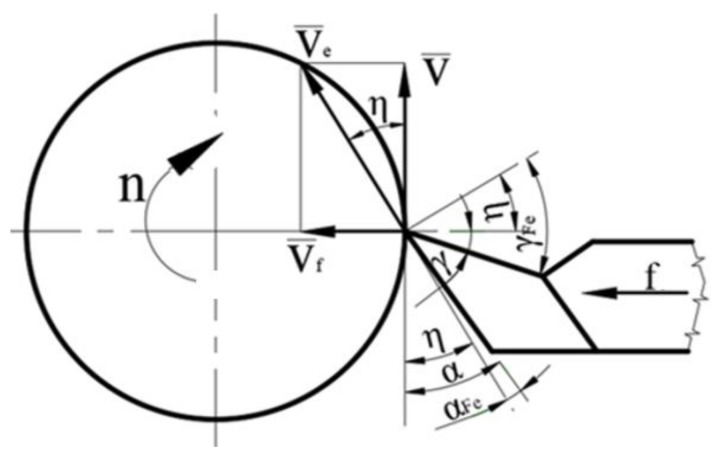
Kinematics of turning machining with transverse feed.

The process of machining by turning with a transverse advance of a part is carried out according to the kinematics shown in Figure 3. Thus, Figure 3 shows that, for the process of machining, two movements are required with speed V for the main movement of cutting, respectively, with the speed *V_f_* for the feed movement. Under these conditions, a resulting cutting motion *V_e_* is obtained. The angle η (angle of the main cutting direction) is formed between the direction of the main cutting motion and the direction of the resulting cutting movement. During the manufacturing process by cutting, the values of the speed V of the main cutting movement vary from a maximum value for the outer diameter of the part and decrease as the diameter to be processed decreases. For these reasons, the value of the angle η is a variable from a minimum on the outside of the part to a maximum value on the inside of the part. The modification of the values of the angle η also determines a change of the clearance angle, obtaining the functional clearance angle γ_Fe_, a modification of the seating angle, and obtaining the functional seating angle α_Fe_. The values of the functional clearance angle of the functional installation angle are determined with the following equations:

γ_Fe_ = γ + η (1)

α_Fe_ = α − η (2)

Regarding the values of the angle η, they can be determined with the help of the relation:

(3) $$tg\eta =\frac{v_{f}}{v_{}}=\frac{n\cdot f}{\pi \cdot D_{M}\cdot n}=\frac{f}{\pi \cdot D_{M}}$$

where: *D_M_* represents the diameter corresponding to a certain point *M* on the surface of the part, *n* is the part′s speed, *f* is the transversal advance of the tool, *V_f_* is the speed of the advance movement, *V* is the speed of the main cutting movement, *η* is the angle of the main cutting direction, *γ* is the clearance angle, and α is the seating angle.

Given Equations (1)–(3), respectively, the values of the functional clearance angle can be determined by means of Equation (4), and by means of Equation (5) that of the functional setting angle:

(4) $$\gamma_{Fe}=\gamma +arctg\frac{f}{\pi \cdot D_{M}}$$

(5) $$\alpha_{Fe}=\alpha -arctg\frac{f}{\pi \cdot D_{M}}$$

As can be seen from Equations (1), (2), (4) and (5), respectively, with the decrease of the diameter to be processed (DM), an increase of the angle η is obtained, and this determines an increase of the functional clearance angle *γ_Fe_* and a decrease of the seating functional angle α_Fe_, with the values obtained being presented in Table 3.

According to the results presented in Table 3, it is observed that the values of the functional angles α_Fe_ and γ_Fe_ change their values very much with the change of the machined diameter, and thus the functional clearance angle α_Fe_ can reach negative values towards the center of the part. This fact makes the cutting process to be carried out in unsuitable conditions have negative effects on the quality of the processed surfaces and their accuracy.

In order to eliminate the negative effects of the variation of the functional geometry, the research aimed to modify the constructive form of the tool used for processing by inserting an elastic disc washer under the removable plate, as shown in Figure 1b (T02), to allow the tool geometry to be maintained at values close to the optimal ones.

### 2.4. Measurement of Vibrations That Occur during the Cutting Process

During the machining operations in the technological system, three types of vibrations occur: forced vibrations, self-vibrations (self-excited vibrations), and relaxing vibrations. The research focused on the analysis of self-vibrations and how they can be reduced.

The occurrence of self-vibrations can be explained based on several theories/hypotheses [26]:

Taylor’s hypothesis states that the occurrence of self-vibrations is determined by the variation of the cutting force during the 4 stages of chip deformation: elastic deformation, plastic deformation, hardening, and breaking.

Kashirin’s theory states that the appearance of self-vibrations is determined by the variation of frictional forces on the tool release face.

Sokolcvsky’s theory states that the cause of self-vibration is determined by the continuous variation of the effective geometric parameters during the irregularities of the processed surface.

Harnis and Grig’s theory states that the appearance of autovibrations is determined by the continuous variation of the cutting depth between the irregularities of the previous processing and the oscillations of the current processing, which determines the appearance of the variation of the cutting force and, implicitly, the appearance of a self-vibrating effect.

Toblas’s theory states that the cause of the self-vibrations is determined by the variation of the cutting forces between the moment when the tool enters the undeformed material and when it is rejected by the hardened material.

**Figure 4:**
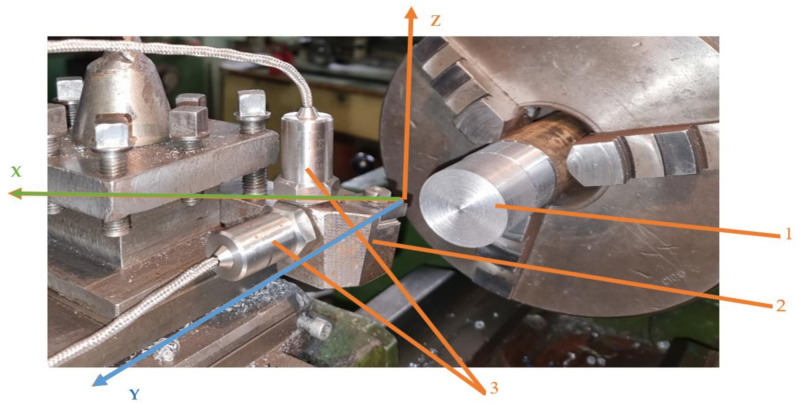
The scheme of the vibration measuring system: 1—the workpiece; 2—the tool; 3—accelerometers for measuring vibrations on Y and Z directions.

The main objective of the research was to ensure constant effective geometric parameters for the cutting tool to reduce the self-vibrations explained by Sokolcvsky’s theory and Kashirin’s theory. Thus, the vibrations that occur during the machining process were measured in two main directions: Z and Y. In this regard, the vibration measurement system shown in Figure 4 was used. Vibrations were measured in two cases, namely rigid fixing of the removable plate on the knife body and elastic fixing, which involves arranging an elastic washer between the removable plate and the tool body so as to obtain constant effective geometric parameters for the cutting tool.

**Figure 5:**
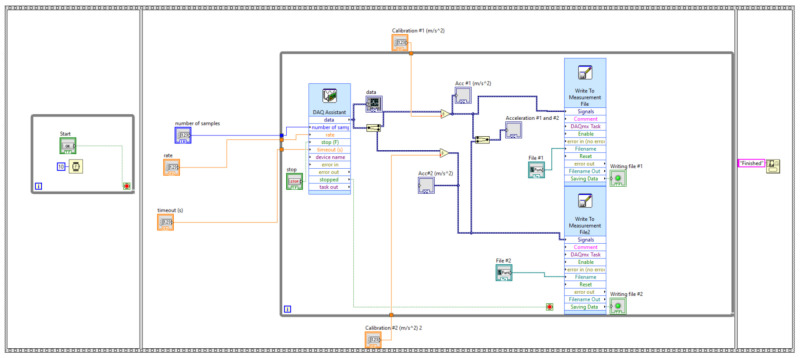
Vibration acquisition and processing tool.

An acquisition tool was designed to take over and process the vibrations that occur during the cutting process, as shown in Figure 5.

Two Monitran MTN/1100C accelerometers with a standard sensitivity of 100 m/g and a frequency response from 2 Hz to 20 kHz were used to measure the vibrations. These were connected to the NI USB-9233 acquisition system. The NI USB-9233 data acquisition device provides a USB interface for four channels of 24-bit analog inputs with integrated signal conditioning. This data acquisition system accepts the connection of IEPE (Integrated Electronics Piezo-Electric) sensors. The NI USB-9233 uses a combination of digital and analog filtering to get the most accurate and noise-free signal possible. Filtering is based on the frequency range or bandwidth of the signal. For the acquisition of the signal from the two accelerometers, we used a virtual instrument, made by the study authors, in the LabView program. The acquisition tool, shown in Figure 5, contains, for both channels used, signal acquisition modules, plotting and saving the signal in ASCII format. Subsequently, the acquired signals were processed to obtain the Fast Fourier Transform (FFT) and the Spectrogram in the Matlab program.

### 2.5. Measurement of the Roughness of the Machined Surfaces

The roughness of the machined surfaces depends on many factors. One that is very important is the geometry of the cutting tool. Thus, the values of the seating and clearance angles greatly influence the roughness of the processed surfaces. In this sense, in the research, the aim was to identify how the two angles influence the roughness of the surfaces. In order to determine the influence of the tool geometry on the surface roughness, we started from a relation that expresses the quantitative dependence between the surface roughness and the cutting speed (in case of turning):

(6) $$V=\frac{C_{v}\cdot K_{v}}{T^{x_{v}}\cdot R_{a}}$$

where T is the durability of the cutting tool, min; *R_a_* is the roughness of the processed surface, µm; and *C_v_*, *k_v_*, *x_v_*, and *y_v_* are coefficients and exponents that depend on the cutting conditions:

(7) $$R_{a}=\frac{C_{v}\cdot K_{v}}{T^{x_{v}}\cdot V}$$

(8) $$R_{a}=\frac{C_{v}\cdot K_{v}}{T^{x_{v}}\cdot V_{f}}\cdot tg\eta$$

(9) $$V_{f}=n\cdot f$$

where *n* is the speed of the part and *f* is the advance of the cutting tool:

(10) $$R_{a}=\frac{C_{v}\cdot K_{v}}{T^{x_{v}}\cdot V_{f}}\cdot tg\left(\gamma_{FE}-\gamma \right)$$

From the analysis of Equation (8), it is observed that the roughness of the surface processed by turning depends on the angle of the main cutting direction *η*. Thus, by making a new constructive variant of the cutting tool, as shown in Figure 1b, the aim is to ensure an optimal value for the angle *η* so that the roughness is maintained at the same value throughout the length of the machined surface.

An ST1 roughness meter provided by Hoffmann Industrial Tools S.R.L., Bucharest, Romania was used to measure the roughness of the surfaces processed by turning. Additionally, the processing of the obtained results was performed using statistical software MINITAB.

## 3. Results and Discussions

Experimental research was conducted in two distinct stages: in the first stage, the vibrations that occurred during the cutting process were measured; in the second stage, measurements were made regarding the roughness of the processed surfaces. In order to obtain adequate results, the measurements related to both vibration and roughness were performed for 10 samples processed under the same conditions. Additionally, the results were established for the case when the cutting tool T01, as shown in Figure 1a, was used for processing, as well as for the case when the cutting tool T02 was used for processing, as shown in Figure 1b.

Vibration measurement was performed by mounting the detecting elements on the body of the cutting tool. The same body type for the cutting tool was used, both for the cutting tool that was the classic construction version and for the improved construction version. In order to be able to perform an analysis of the correlation between the vibrations that appear during machining and the roughness of the machined surface, statistical processing of the experimental results was performed using the MINITAB software.

### 3.1. Analysis of Vibrations That Occur during the Manufacturing Process by Cutting

**Figure 6:**
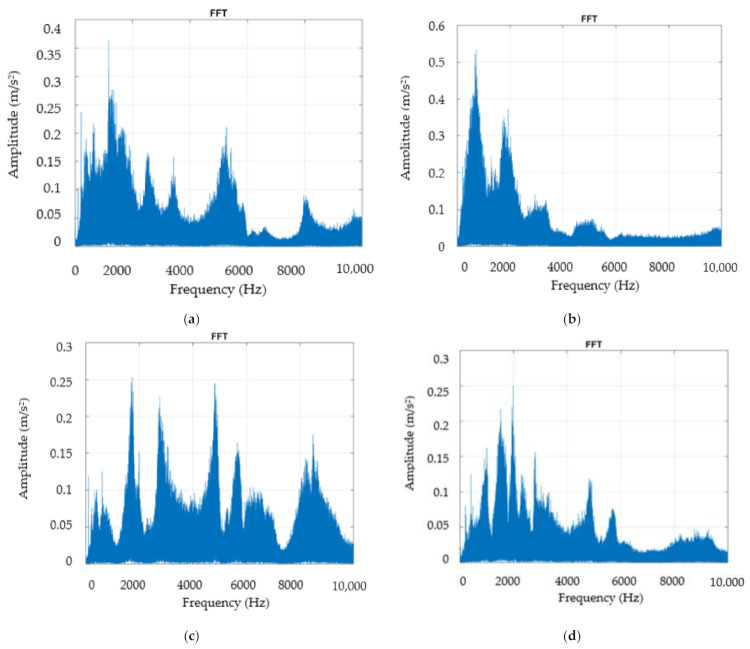
Vibration analysis by applying FFT: (**a**) in the Z direction, in the case of machining with a cutting tool T01; (**b**) in the Y direction in the case of machining with a cutting tool T01; (**c**) in the Z direction in the case of machining with the cutting tool T02; (**d**) in the Y direction in the case of machining with a T02 cutting tool.

The mechanical assembly consisting of a cutting tool, a workpiece, or a machine tool forms a mechanical system of such a nature that the representative vibrations appear only at low frequencies measured in the range 10–1000 Hz [27,28]. One of the important characteristics of vibrations is the amplitude of the vibration and the characteristic that describes the severity of the vibration and can be quantified in several ways. Thus, on the vibration diagram, one can observe the relationship between the peak-to-peak level, the peak level, and the average level of a sinewave. The value of the peak-to-peak level is very important in that it indicates the maximum amplitude of the wave determined by a certain maximum load. The value of the peak-to-peak level is particularly valuable for indicating the level of short-term shocks, etc. The rectified mean value (RMS), on the other hand, takes into account the time history of the vibration but is considered of limited practical interest. However, the RMS value is the most relevant measure of the amplitude, as it takes into account the evolution of the wave over time and provides a value of the amplitude that is directly related to the energy content and the destructive capabilities of vibration. In the research conducted, the analysis of vibrations in the system was performed in the first stage using Fast Fourier Transform (FFT), which is a method of analysis based on the waveform of vibrations. Because waveforms are generally complicated and difficult to analyze by applying FFT, they can decompose into a series of discrete sine waves. Thus, the FFT analysis took into account the vibrations on the two directions Z and Y, respectively, both for the machining processes performed with the cutting tool T01, and for those performed with the use of the cutting tool T02. The experimental results obtained in the case of FFT application are presented in Figure 6.

From the analysis of the results obtained by applying FFT, as presented in Figure 6, the values in the frequency range 10–1000 Hz were mainly retained due to the fact that the representative vibrations for such a mechanical system occur in this range. Thus, in the case of the FFT analysis in the Z direction for the case where the cutting tool T01 (Figure 6a) and the cutting tool T02 (Figure 6c) were used during the processing, the following were observed:

- In the Z direction, when using the cutting tool T01, the vibration amplitude has a maximum value of about 0.35 m/s^2^, and when using the cutting tool T02, the vibration amplitude has a maximum value of about 0.12 m/s^2^, comparable to the minimum value obtained for the situation where the processing is made with the cutting tool T01. Thus, the use of the tool T02 determines a reduction of the amplitude of vibrations of approximately 3 times. This can be explained by the fact that the use of the cutting tool T02 determines the maintenance of an optimal geometry of the cutting tool, especially regarding the value of the angle α_Fe._ Under these conditions, the friction on the face of the cutting tool is greatly reduced, with consequences on the amplitude of vibrations;
- From the analysis of the amplitude of vibrations in the Y direction, when using the cutting tool T01, it was observed that it has a maximum value of about 0.52 m/s^2^, and when using the cutting tool T02, the amplitude of vibrations has a maximum value of about 0.16 m/s^2^ less than the minimum value for the situation in which the processing is performed with the cutting tool T01. Under these conditions, the use of the tool T02 determines a reduction of the amplitude of vibrations of approximately 4 times. These results demonstrate that the use of the T02 cutting tool has a greater effect on the reduction of Y-direction vibrations than on the Z direction, and this can be explained by the fact that the use of the T02 cutting tool reduces the friction between the workpiece material and the clearance face of the tool, by maintaining optimal values for the functional seating angle of the tool α_Fe._

All these results confirm that the use in machining of tools with improper geometry causes the appearance of vibrations in the technological system, with negative effects on the precision of machining and the roughness of the surfaces of the machined parts [29].

Because the analysis of vibrations using only the FFT method does not allow the best results to be obtained, in the next stage of research, the Short-Time Fourier-Transformation (STFT) method was used. This provides a more conclusive image, because it also considers the analysis of vibrations in time function. Spectrogram analysis is a viable solution for extracting information on how vibrations evolve. The use of the STFT method offers advantages due to the fact that a complete vibration analysis cannot always be performed only by tracking stationary signals in a certain frequency range. The main difficulty with transient signals is that the FFT method is not able to represent the time behavior of vibrations. STFT is based on the Discrete Fourier Transform (DFT), which represents the frequency and phase components of a section of a time-dependent signal. In order to obtain the best results for the vibration signal, in addition to the high sampling frequency of the time signal, it is also necessary to optimize the resolution according to time and frequency [30,31].

During the turning by cutting the piece in a rotational motion, it was decided to obtain a high-frequency resolution in a time segment for the creation of spectrograms with the application of the overlapping window technique. During the analysis, it was assumed that the time segments are narrow enough to be considered as quasi-stationary segments.

**Figure 7:**
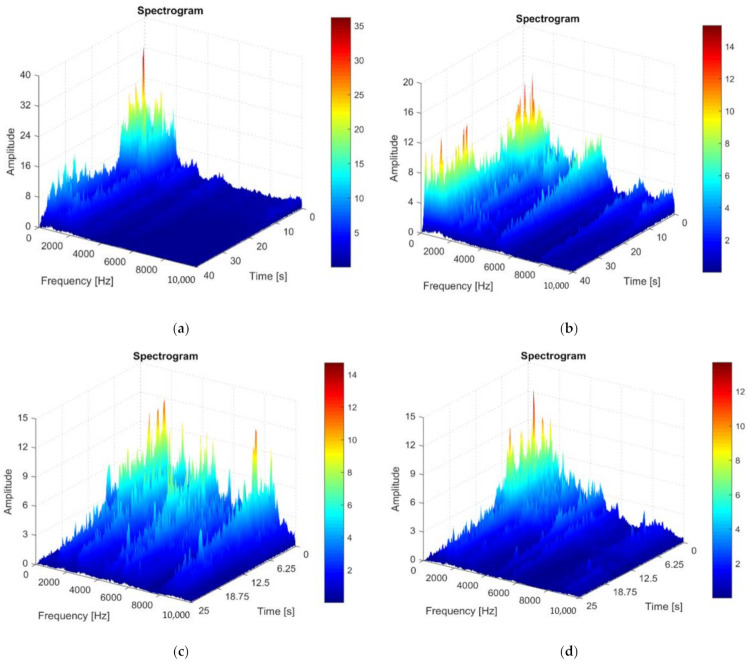
The spectrogram of vibration recorded during machining: (**a**) in the Z direction in the case of machining with tool T01; (**b**) in the Y direction in the case of machining with the cutting tool T01; (**c**) in the Z direction in case of machining with the cutting tool T02; (**d**) in the Y direction in the case of machining with a T02 cutting tool.

The vibration spectrograms were made for the case when the tool T01 is used in the processing (Figure 7a—direction Z, Figure 7b—direction Y), but also in the case where the tool T02 is used (Figure 7c—direction Z, Figure 7d—Y direction).

The analysis of the spectrograms presented in Figure 7 was performed considering the entire frequency range 10–1000 Hz. Additionally, from the analysis of the spectrograms, it is possible to very easily observe the evolution in time of the amplitude of vibrations, and this offers an image regarding the size of the amplitude of vibrations depending on the processed diameter.

Thus, from the analysis of the spectrograms, it was observed that the highest amplitude of vibrations occurs when using the cutting tool T01 in the Z direction. This can be explained by the fact that the cutting forces have the highest values in the Z direction and thus, in this direction, the highest friction forces and implicit vibrations with the highest amplitude can occur. It is also worth mentioning that the amplitude of vibrations, in the Z direction, at the beginning of the processing process was in the case of using the cutting tool T01 was 32 m/s^2^ compared to the situation when the cutting tool T01 was used when it was 12.5 m/s^2^. Thus, it could be observed that the use of the T02 cutting tool determines a reduction of the amplitude of vibrations, in the Z direction, by about 2.5 times. Regarding the amplitude of the vibrations on the Y direction, at the beginning of the processing process, a maximum amplitude of approximately 16 m/s^2^ in the case of using the tool T01 and 12 m/s^2^ in the case of using the tool T02 was observed.

The analysis of the spectrograms shows that, at the end of the machining process, the amplitude of vibrations decreases substantially compared to the beginning of the machining process, both in the Z and Y direction when using tool T02 but remains at considerably higher values when using tool T01. This can be explained by the fact that the use of the T02 tool allows for damping of vibrations that may occur in the cutting process. It should be noted that the use of the T02 cutting tool determines a very small value of vibrations towards the end of the processing process, i.e., the vibration amplitude tends to 0. Additionally, maintaining high and approximately equal values of the vibration amplitude in the Y direction, both at the beginning of processing as well as at the end of it, when using the T01 tool (Figure 7b) can be explained by the fact that the friction between the tool seating face and the workpiece material remains high throughout the machining process. Thus, it is confirmed that the analysis of vibration spectrograms provides the most useful information that can be used to adopt constructive solutions to reduce the amplitude of vibrations in a mechanical system [32].

**Figure 8:**
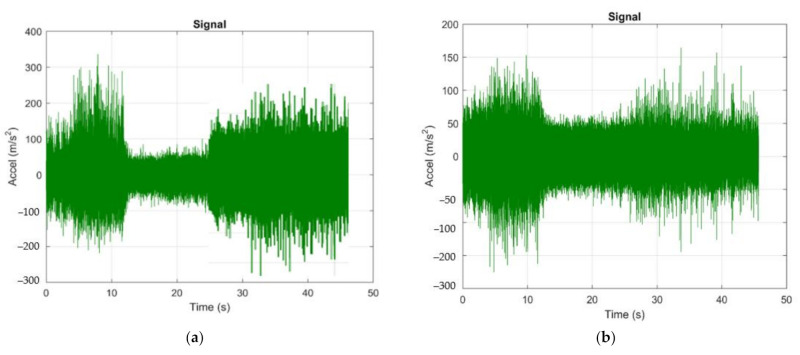

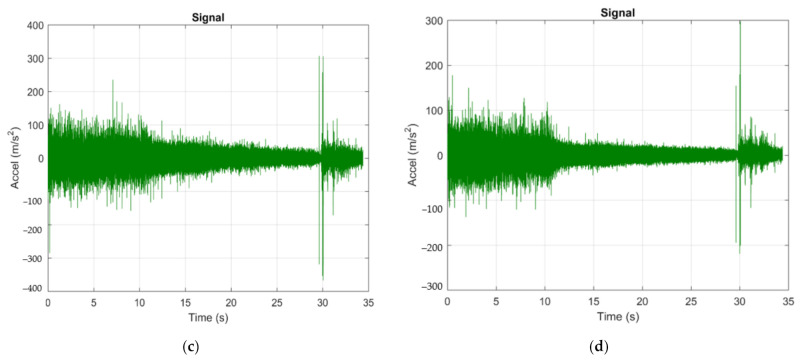
Acceleration of vibrations recorded during machining: (**a**) in the Z direction in the case of machining with a cutting tool T01; (**b**) in the Y direction in the case of machining with a cutting tool T01; (**c**) in the Z direction in the case of machining with a T02 cutting tool; (**d**) in the Y direction in the case of machining with a cutting tool in variant T02.

Because the vibration parameters change over time, signal analysis is important, which gives us the dependence of the acceleration vibration as a function of time. Thus, in order to obtain a better analysis of the vibrations that occurred during the cutting process in the next stage of research, the aim was to determine the acceleration of vibrations as a function of time, as shown in Figure 8. This analysis of vibrations as a function of time may allow monitoring of the vibration levels. During the vibration analysis, the acceleration was measured in m/s^2^, but it can also be measured in G (1G = approximately 9.8 m/s^2^). Acceptable operating vibration limits may be predefined, or certain standards may be referred to.

Figure 8 shows the acceleration vibration signals obtained in the two directions, Z and Y, respectively, for both types of tools (T01 and T02). From the analysis, it was observed that the signal itself is totally different for the case when the tool T01 and the tool T02 are used. From the analysis of the vibration amplitude over time, it was observed that the maximum value of acceleration was higher when using the cutting tool T01 (Accel = 300 m/s^2^) compared to if the cutting tool T02 was used for processing when the value of acceleration was Accel = 200 m/s^2^. It should be noted that if the cutting tool T01 acceleration vibration was used, a low value was observed in the middle of the processing time and a maximum value at the beginning and end of the processing. As for vibration acceleration, when using the T02 cutting tool, it had a high value at the beginning of the machining process, after which it gradually decreased until the time of 30 s. After this time, the acceleration vibration value increased sharply for a short period of time, and this can be explained by the fact that, after a period of 30 s, the actual processing of the material was completed (given the dimensions of the processed surface and the parameters cutting regime), and after this time the tool was removed from the cutting.

The results obtained and presented in Figure 8 confirm that the geometry of the cutting tool has a very large influence on the vibrations that occur during the cutting process [33]. Thus, incorrect geometry of the cutting tool can cause an increase in the frictional forces between the tool face and the workpiece material and, implicitly, the acceleration vibration. The use of a T02 cutting tool for machining allows the maintenance of an optimal functional geometry throughout the machining process. Research has also shown that the presence of an elastic element in the structure of the cutting tool allows both vibration damping and permanent adjustment of its position so as to avoid an excessive decrease in the value of the angle α_Fe._ According to the results presented in Table 3, the angle αFe has very small or even negative values, as the processing advances towards the center of the part. Under these conditions, the use of the T02 tool is a viable solution that can reduce the size of vibrations, and this has positive effects on the accuracy and quality of machined surfaces.

### 3.2. Analysis of the Roughness of the Surfaces Processed by Cutting

The surface roughness is important for the part because it is directly related to its functional role. Therefore, the investigation of the surface roughness of the part is essential in order to be able to determine how it behaves in operation [34]. Currently, machines operate at higher speeds and loads, which require greater dimensional and geometric accuracy, along with the surface quality of the finished parts. The ability of a manufacturing process to allow the desired roughness to be obtained depends on many parameters, but the construction of the cutting tool has a great influence. Thus, the main purpose of the research was to design a constructive form of the cutting tool that would allow the best roughness for the processed surfaces to be obtained. In this sense, the cutting tools T01 and T02 presented in Figure 1 were designed, made, and used in the research.

In the first stage of the research, an analysis of the vibrations that occur during the cutting process was performed and the two types of tools T01 and T02 were used. This analysis was necessary because the vibrations that occur during cutting substantially influence the roughness of the machined surface. The aim of the T02 tool was to ensure an optimal functional geometry for the cutting tool that would allow the partial elimination of vibrations that occur in the cutting process because the vibration components particularly influence the quality of the machined surface.

**Figure 9:**
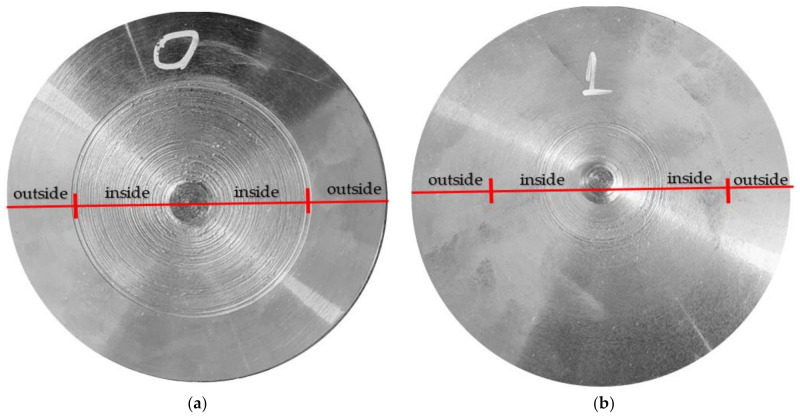
Images with surface roughness obtained by machining: (**a**) in the case of machining with a T01 cutting tool; (**b**) in the case of machining with a T02 cutting tool.

In order for the results obtained during the experimental research to be relevant, 10 samples for which the roughness was measured were processed under the same conditions. The roughness of the obtained surfaces was measured for two distinct areas, namely an area arranged towards the outside of the part and an area arranged towards the inside of the part. The measured roughness values are shown in Table 4, and an image with the shape of the obtained roughness, for sample number 6, is shown in Figure 9. The presentation of the image of sample number 6 was established considering that it had the highest roughness for the surface arranged to the outside of the piece.

The surface roughness of the parts obtained when using the two tools, T01 and T02, was very different, as shown in Figure 9. This difference in roughness was very large, especially towards the center of the part, where the roughness of the workpiece with tool T01 is very high while the roughness obtained with the T02 tool is much lower. Thus, from the analysis of the results presented in Table 4, it was observed that the surface roughness measured towards the outside of the surface in the case of using the two tools T01 and T02 is quite small but higher in the case of using tool T01. These roughness values are thus correlated with the values obtained in the vibration analysis stage, as shown in Figure 6, Figure 7 and Figure 8.

Very large differences regarding the surface roughness were obtained towards the inside of the part (Figure 9, Table 4). Thus, if the tool T01 was used for processing, the roughness Ra was about 6 times higher than if the tool T02 was used. This is also explained by the fact that the amplitude of the vibrations was very high towards the center of the part when the tool T01 was used, as shown in Figure 8a,b, and if the tool T02 was used, the amplitude of vibrations had very small values towards the center of the part, as shown in Figure 8c,d. Although the roughness towards the inside of the obtained surface when the tool T02 was used was quite low, its value was slightly higher than on the outside of the part, and this can be explained by the fact that the optimized shape of the elastic disc washer, used in the construction of the T02, was not achieved. However, the results obtained are remarkable considering that towards the inside of the part, the reduction of roughness obtained in the case of using the tool T02 is approximately 4 times smaller than the situation when T01 was used.

**Figure 10:**
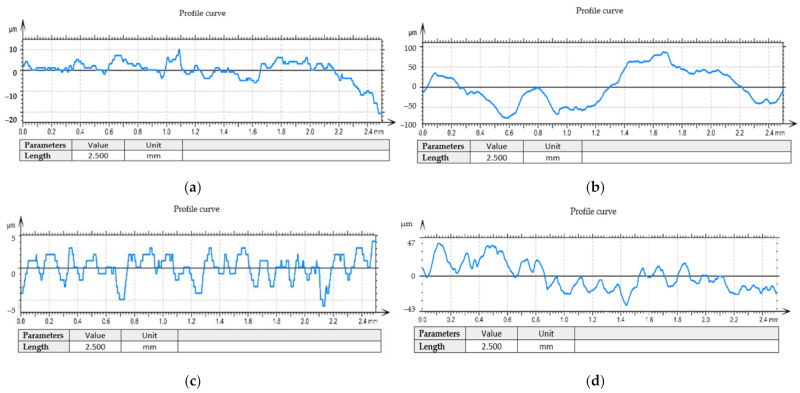
Profile curve: (**a**) in case of machining with the cutting tool T01 to the outside; (**b**) in case of machining with a T01 cutting tool inwards; (**c**) in case of machining with a T02 cutting tool to the outside; (**d**) in case of machining with a T02 cutting tool inwards.

**Figure 11:**
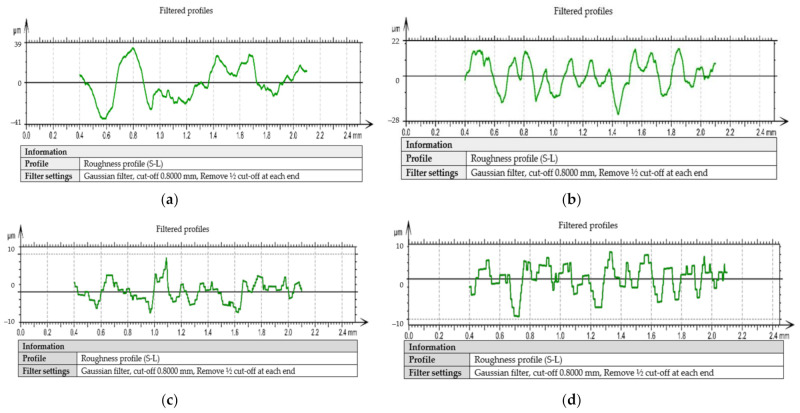
Filtered profiles: (**a**) in case of machining with the cutting tool T01 to the outside; (**b**) in case of machining with a T01 cutting tool inwards; (**c**) in case of machining with a T02 cutting tool to the outside; (**d**) in case of machining with a T02 cutting tool inwards.

For the test tube number 6, an analysis of the surface profiles was performed, as shown in Figure 10 and Figure 11. The quality of the processed surface is reflected very well by the curves of the surface profile. The surface profiles were obtained taking into account the coordinates for measuring the surface roughness (Y, Z). Thus, the following was found for the analyzed sample:

- When using the tool T01, the profile of the curve for the outside, as shown in Figure 10a, is relatively stable within ±10 µm, but as we move away from the surface of the part, the profile of the curve becomes unstable and reaches very high values, towards −30 µm;
- The profile of the curve, in case of using the tool T02, for the outside, as shown in Figure 10c, is a very stable one within the limits ± 5 µm, being much smaller compared to the situation of using the tool T01;
- For the surface arranged towards the inside of the part, it was found that in the case of using tool T01, the curve profile is very unstable, falling within the limits ± 100 µm, and when using the T02 tool, the curve profile becomes stable after a cutting distance of approximately 1.5 mm, this being approximately within the limits of ± 20 µm.

During the processing of the obtained experimental data, a series of curves related to filtered profiles were drawn, as shown in Figure 11, and these curves also highlight the advantage offered by the use of the T02 tool for the roughness of the processed surfaces. Filtered profiles eliminate wavelengths outside a band of interest, and the wavelengths within the band of interest are weighted by the mathematical properties of the roughness analysis algorithm. Additionally, the roughness values obtained confirm the direct link between the vibrations that occur during the cutting process and the roughness of the machined surfaces [31].

**Figure 12:**
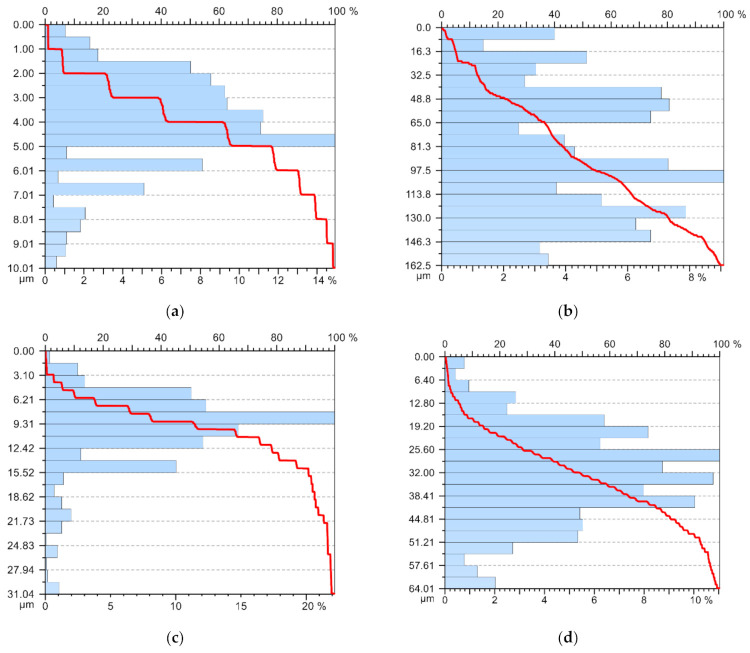
Abbott–Firestone curve: (**a**) in case of machining with the cutting tool T01 to the outside; (**b**) in case of machining with a T01 cutting tool inwards; (**c**) in case of machining with a T02 cutting tool to the outside; (**d**) in the case of machining with a T02 cutting tool inwards.

The roughness values obtained for the 10 samples were recorded and processed, obtaining the graphs presented in Figure 12. From their analysis, it was observed that the roughness values had quite stable values if the tool T02 was used, and in the case in which the tool T01 was used, the stability of the roughness values was not adequate.

The experiments showed that tools with improved construction (T02) allow the realization of a ductile-machining regime, and this, according to [35,36,37,38], can cause a reduction in the amplitude of vibrations and thus the roughness of surfaces. Additionally, the use of the spring washer allows the tool geometry to be modified adaptively according to the machining conditions, and this determines that a surface without cracks and a high machining efficiency can be obtained.

## 4. Conclusions

This research has shown that the self-vibrations that occur during the cutting process substantially influence the quality of the processed surfaces. Thus, the studies considered the control of self-vibrations by making a T02 tool, which implies an improved constructive variant in relation to the classic constructive variant used for the T01 cutting tool. The use of the two types of tools for turning with a transverse feed demonstrated the following:

- The use of the T02 tool determines a reduction of the amplitude of approximately 3 times compared to the situation in which the T0 tool was used. This can be explained by the fact that, when using the T02 tool, the friction on the seating face or the tool clearance face decreases a lot, with consequences on the amplitude of vibrations;
- The vibration analysis using the FFT method and STFT allowed an in-depth analysis of the vibration amplitude for a frequency of up to 1000 Hz, which showed that the vibration amplitude is kept quite high over the entire stroke of the tool T01, and, in the case when using the T02 tool, the amplitude of the vibrations decreases considerably as the center of the part is approached;
- The analysis of the quality of the workpiece surface showed that if the T01 tool was used, the roughness of the machined surface is quite high and has a tendency to increase sharply as it approaches the center of the workpiece;
- The use of the T02 tool allows an approximately constant roughness over the entire surface to be obtained, without large differences in the roughness between the surfaces arranged towards the inside or outside of the part.

Research has demonstrated the advantages of using the T02 tool in terms of vibration control and, implicitly, on the quality of machined surfaces. Future research will follow the use of the constructive variant of the T02 tool for processing and other types of materials but also the optimization of the constructive form of the cutting tool T02, depending on the specific conditions that characterize each cutting process.

## Figures and Tables

**Table 1:** Chemical composition of steel C45 (1.0503), %.

Cr + Mo + Ni = max 0.63							
C	Si	Mn	Ni	P	S	Cr	Mo
0.43–0.5	max. 0.4	0.5–0.8	max. 0.4	max. 0.045	max. 0.045	max. 0.4	max. 0.1

**Table 2:** Mechanical properties of steel C45 (1.0503) in the normalized state.

Diameter d, [mm]	<16	>16–100	>100–250
Thickness t [mm]	<16	16 < t < 100	100 < t < 250
0.2% proof stress R_p0.2_, [N/mm^2^]	min. 340	min. 305	min. 275
Tensile strength R_m_, [N/mm^2^]	min. 620	min. 580	min. 580
Fracture elongation A_5_, [%]	min. 14	min. 16	min. 16

**Table 3:** The values of the angles *η*°, *α_Fe_*°_,_ and *γ_Fe_*° depending on the processed diameter (*D*_M_).

The Advance f (mm/rot)	0.36						
Diameter (mm)	150	120	90	60	30	15	1
η°	0.043	0.054	0.072	0.109	0.218	0.437	6.53
α_Fe_°	5.957	5.946	5.928	5.891	5.782	5.563	–0.53
γ_Fe_°	5.043	5.054	5.072	5.109	5.201	5.437	11.53

**Table 4:** The measured roughness values for the 10 samples, *Ra* (µm).

The Sample Number	Outside		Inside	
	In the Case of Machining Made with the Cutting Tool T01	In the Case of Machining Made with the Cutting Tool T02	In the Case of Machining Made with the Cutting Tool T01	In the Case of Machining Made with the Cutting Tool T02
1	2.67	1.64	12.50	3.71
2	3.30	1.67	12.42	3.17
3	2.97	1.68	12.79	2.47
4	3.28	1.58	12.40	3.64
5	2.91	1.87	12.61	3.46
6	3.22	1.90	12.62	3.62
7	2.96	1.65	12.23	3.23
8	3.06	1.40	12.77	3.26
9	3.19	1.89	12.74	3.17
10	3.07	1.50	12.93	3.28
Mean	3.06	1.68	12.60	3.40
St Dev	0.19	0.16	0.21	0.20
Cvariation	6.37	10.01	43.64	7.59
Median	3.06	1.66	12.61	3.37
*p*-value	0.56	0.36	0.856	0.19
